# Dynamic manipulation of particles via transformative optofluidic waveguides

**DOI:** 10.1038/srep15170

**Published:** 2015-10-16

**Authors:** Kang Soo Lee, Kyung Heon Lee, Sang Bok Kim, Byung Hang Ha, Jin Ho Jung, Hyung Jin Sung, Sang Soo Kim

**Affiliations:** 1 Department of Mechanical Engineering, KAIST 291 Daehak-ro, Yuseong-gu, Daejeon 34141, Korea

## Abstract

Optofluidics is one of the most remarkable areas in the field of microfluidic research. Particle manipulation with optofluidic platforms has become central to optical chromatography, biotechnology, and μ-total analysis systems. Optical manipulation of particles depends on their sizes and refractive indices (*n*), which occasionally leads to undesirable separation consequences when their optical mobilities are identical. Here, we demonstrate rapid and dynamic particle manipulation according to *n*, regardless of size. Integrated liquid-core/solid-cladding (LS) and liquid-core/liquid-cladding (L^2^) waveguides were fabricated and their characteristics were experimentally and theoretically determined. The high and low *n* particles showed the opposite behaviors by controlling the contrast of their *n* values to those of the working fluids. The LS waveguide was found to successfully manipulate particles according to *n*, and the L^2^ waveguide was found to provide additional system stability and flexibility, compared to the LS system.

Research into the combination of optics and fluidics, namely optofluidics, has been extensive over the past few decades^1^,^2^,^3^,^4^,^5^. Its history began with the use of mercury in a liquid mirror telescope, and thereafter a broad window for research topics was opened by combining of microfluidics and photonics^6^. To date, a large range of optofluidic systems have been reported, including reconfigurable optical components (e.g., mirrors, lenses, filters, and waveguides), sensors using specific optical configurations (e.g., whispering gallery mode (WGM), surface-enhanced Raman scattering (SERS), and total internal reflection fluorescence microscopy (TIRFM)), and energy applications (e.g., optofluidic algae cultivation systems)^7^,^8^,^9^,^10^,^11^,^12^,^13^,^14^,^15^,^16^,^17^. Meanwhile, in the area of photonics, there have been many developments in transformation optics, which have enabled the generation of spatially variable electromagnetic characteristics such as permittivity and permeability^18^. In particular, several studies have reported the use of optofluidics as transformation optics. For example, Yang *et al.*^19^ demonstrated light manipulations including self-focusing and interference with optofluidic waveguides at low flow rates and Morton *et al.*^20^ tested an optofluidic metamaterial that uses a deterministic lateral displacement (DLD) array.

Since the first light waveguide was reported in a study of a water fountain in the early 1840s, they have been widely used in research and industry as light guides and communication tools^21^. In particular, optofluidic waveguides, which utilize liquids as the medium for light delivery, have many advantages over conventional solid-core/solid-cladding waveguides (i.e., optical fibers): (1) their optical characteristics can be dynamically controlled by simply altering the working fluids, (2) in sensor applications the target analytes or particulate materials can travel in the working fluids, and (3) the interface between the core and cladding liquids is smooth optically and fluid-dynamically when two miscible fluids are used^22^,^23^. Thus, optofluidic waveguides have attracted extensive research attention, even though they exhibit one significant weakness in communication technology applications, namely higher attenuation losses of ~1 dB/cm, in contrast to those of optical fibers, ~0.2 dB/km^22^,^24^.

Particle manipulation with optical radiation forces has been studied in order to realize precise and discretionary particle control at the single particle level in the areas of life and environmental sciences, microbiology, and microecology since Ashkin’s pioneering work^25^,^26^,^27^,^28^. There are two types of particle manipulation: tweezer and separator. In general, a tweezer confines a single particle inside a laser beam region by using an optical radial force, otherwise known as an optical gradient force^25^. In a separator with a fluidic system configuration, on the other hand, the particle is not captured but just passes through the laser beam region, which means that its lateral position is changed by the optical force and the particle of interest is separated from the sample stream^29^,^30^,^31^. Separation characteristics depend on both the refractive index (*n*) and the size of the particle, so particles can exhibit similar separation characteristics when their optical mobilities, which combine *n* and particle size into a single parameter, are comparable even though their sizes and *n* values are different^32^,^33^. Further, separation does not enable rapid analyzing and sorting because the optical force is weak (~pN)^34^,^35^,^36^.

We provide here a de facto demonstration of rapid and dynamic particle manipulation according to their *n* values, regardless of particle size, with transformative optofluidic waveguides. Two different system configurations were tested: liquid-core/solid-cladding (LS) and liquid-core/liquid-cladding (L^2^) waveguides. Several types of optofluidic waveguide systems have been reported based on either total internal reflection (TIR) or interference^37^,^38^. We used a TIR-based platform, which ensures structural simplicity and versatility. When the core fluid has a higher *n* value than either the solid or the liquid cladding, the light is guided along the core fluid. Figure 1 shows a laser beam propagation in the LS system. The channel is composed of the widely used organic polymer, polydimethylsiloxane (PDMS; *n* = 1.41). Light is guided along the fluidic channel by TIR when benzyl alcohol (*n* = 1.54; see Fig. 1(b)) is used as the core fluid, whereas it propagates freely along the channel filled with deionized (DI) water (*n* = 1.33; see Fig. 1(a)). Several candidate working fluids were evaluated; their properties are presented in the supplementary material.

## Results

### Liquid-core/solid-cladding (LS) waveguide

**Figure 2:**
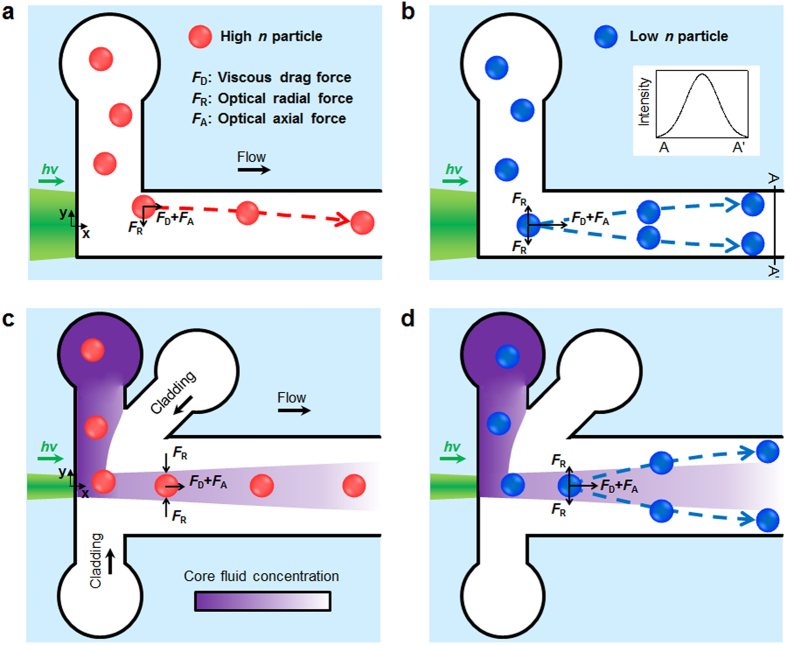
Operational principles of the two types of particle manipulation systems: trajectories of the (**a**) high and (**b**) low *n* particles in a liquid-core/solid-cladding (LS) design. The high *n* particle is pulled into the center of the channel by the positive optical radial force, and the viscous drag force and the optical axial force drive the particle along the flow direction. The low *n* particle, in contrast, is pushed away into the side wall by the negative optical radial force. The inset of (**b**) shows the Gaussian beam intensity distribution in the lateral direction (A–A′). Trajectories of the (**c**) high and (**d**) low *n* particles in a liquid-core/liquid-cladding (L^2^) design which is formed by a hydrodynamic flow focusing geometry. The purple region represents the core fluid concentration; the waveguide fades along the flow direction due to diffusion between the core and cladding fluids.

Figure 2 shows the operational principles of the two different waveguide systems. In the LS system as shown in Fig. 2(a), when a high *n* particle (a particle with *n* higher than that of the surrounding medium) is injected into the LS waveguide region, it moves to the center of the microchannel due to the positive optical radial force. In contrast, the negative optical radial force acting on a low *n* particle (a particle with *n* lower than that of the surrounding medium) liberates it from the optical axis into the flank region as shown in Fig. 2(b). The laser intensity distribution in the lateral direction when a Gaussian beam is used is portrayed in the inset. As a result, particles are manipulated according to their *n* values, regardless of their sizes, which means that the threshold dividing the opposite particle behaviors can be dynamically controlled by adjusting the *n* value of the working fluid. Once the particles are manipulated, the pinched region extends the lateral distance between high and low *n* particles^39^ (more details can be found in the supplementary material).

**Figure 3:**
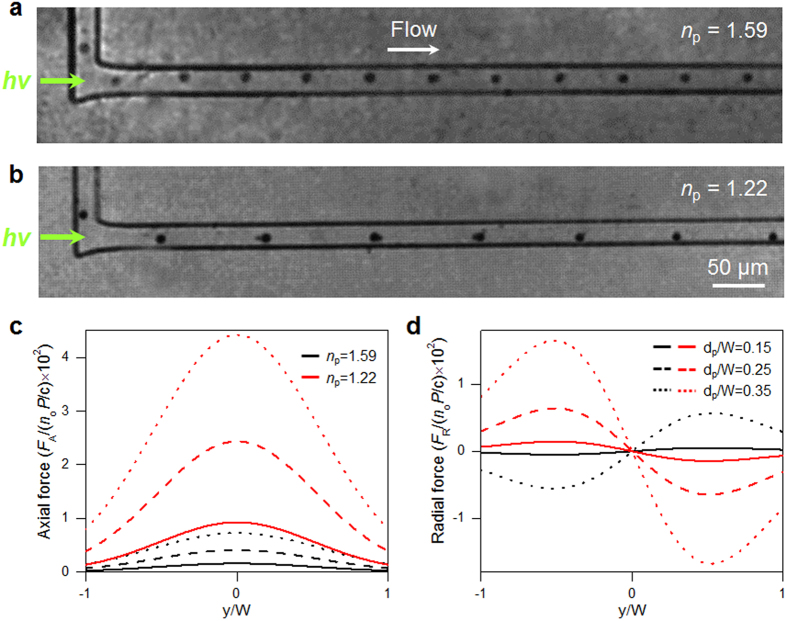
Results for the LS waveguide system: trajectories of (**a**) high (d_*p*_ = 5 μm) and (**b**) low *n* (d_*p*_ ~ 5 μm) particles, numerical calculations of (**c**) optical axial and (**d**) radial forces on these particles. The labels of (**c**,**d**) are mutual.

Figure 3(a,b) show the trajectories of high and low *n* particles in the LS system. Polystyrene latex (PSL; *n* = 1.59) and hollow glass (*n* = 1.22) particles were used as the high and low *n* particles respectively, and dimethyl sulfoxide (DMSO; *n* = 1.48) was used as the working fluid. As described above, the particles showed the opposite behaviors according to the contrast between their *n* values and those of the surrounding medium. In order to make horizontal particle motion dominant over vertical motion, the channel width was designed to be narrower than the channel depth. The laser intensity distributions in the horizontal (*y*-) and vertical (*z*-) directions were numerically scrutinized for various channel aspect ratios (see the supplementary material). In the present design, (1) the system achieves particle manipulation only according to *n*, regardless of particle size, (2) the behavior of high and low *n* particles depends on the working fluid, (3) the region of optical radial force can be elongated compared to that in illumination systems because it utilizes a guided beam, (4) vertical focusing is not needed, and (5) it sustains label-free and continuous manipulation strategy. The optical radiation forces on each particle were calculated with the geometrical optics approach, specifically the photon stream method^40^. Every photon interacting with the microsphere was traced and the net effects of its refraction and reflection were accounted for in the calculations (for details, see the Methods section). In this system, the optical axial force accelerates the particle motion along the beam propagation direction (i.e., the flow direction) – this effect diminishes as the flow velocity increases – and the optical radial force has different effects on the high and low *n* particles: the sign of the radial force on the high *n* particles is opposite to that on the low *n* particles, whereas the axial forces on the two types of particles are both positive and differ only in magnitude. The magnitudes of the axial and radial forces are proportional to the particle diameter, *d*_p_.

**Figure 4:**
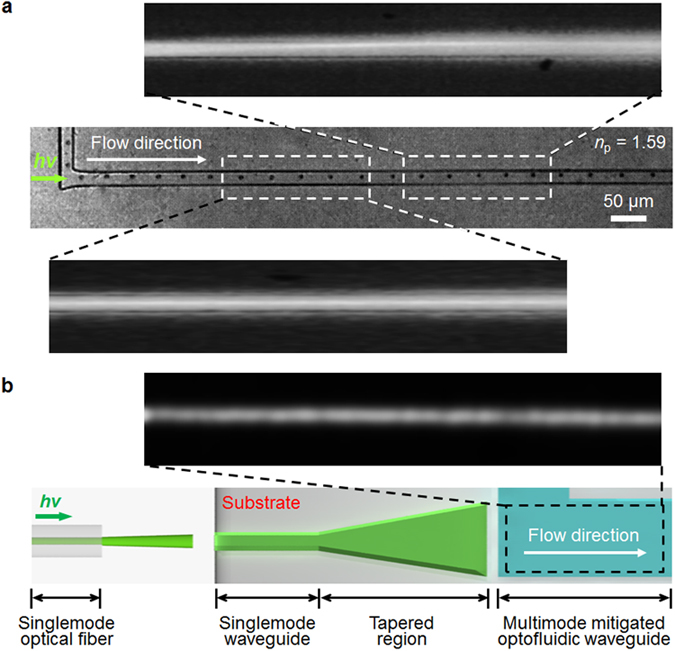
Particle fluctuation in the LS system due to the multimode effect: (**a**) variation in the intensity distribution along the flow direction and the consequent particle fluctuation. (**b**) Multimode-mitigated channel geometry and its intensity distribution. First, a single mode optical fiber is coupled to the single mode waveguide. Second, the light encounters the tapered region for the geometrical expansion and travels along the optofluidic waveguide. The inset shows the measured optical intensity distribution along the channel.

The stability of the LS system is affected by intrinsic features. Figure 4 shows the system instability that arises because of the multimode effect. When the light has an undesirable incident angle or even when it is precisely coupled with the optofluidic waveguide, there is a possibility that a high *n* particle, which is focused at the center of the channel, will fluctuate into the sidewall due to the multimode effect^41^,^42^. In order to visualize the intensity distribution along the channel, rhodamine B (Sigma Aldrich, USA) was dissolved in the working fluid (DMSO) and images were obtained. As shown in Fig. 4(a), the high *n* particle (d_*p*_ = 5 μm) is focused at the center of the channel when the lateral intensity distribution is the same as that of the incident laser beam, i.e., a Gaussian shape. However, as it passes along the channel and encounters the abnormal intensity distribution region due to the multimode effect, it is pushed away into the sidewall, as are the low *n* particles. By the same token, the high *n* particle moves to the sidewall when it is first introduced into the optical force influence region (Figs 3(a) and 4(a)), since the laser beam was coupled to the LS waveguide facing slightly toward the sidewall. To mitigate the multimode effect, a tapered waveguide has been proposed that could resolve the issues in our system (Fig. 4(b))^42^,^43^. However, higher optical losses result from the additional geometry, and there are limitations on the height of the main fluidic channel because this geometry is able to resolve the issue only in two dimensions, which hinders the versatility of this approach^43^. Moreover, although the LS waveguide system is easily fabricated as a single liquid enclosed by a PDMS channel, a perfectly seamless PDMS wall cannot be prepared with conventional soft-lithography processes, which is likely to result in optical losses between the channel wall and the core fluid.

### Liquid-core/liquid-cladding (L^2^) waveguide

In order to resolve the aforementioned shortcomings, we tested a liquid-core/liquid-cladding (L^2^) waveguide system for particle manipulation; its principles of operation are shown in Fig. 2(c,d). In a similar manner to the LS system, particles either focus or diverge depending on their *n* contrast with the core fluid. A pinched region is present beyond the optical forcing region to improve the lateral distance between the high and low *n* particles. Simply using a liquid as a cladding overcomes several constraints on the LS system: (1) even with the spatial disturbance of the Gaussian intensity distribution due to the multimode effect, the L^2^ system does not suffer from fluctuating particle motions because once the low *n* particle is transferred into the cladding fluid, the laser beam does not affect its motion. (2) Irrespective of the spatial/temporal coherences of the laser beam, once the particles respond to the optical force according to their *n* values, they do not interact with the laser beam because the waveguide fades away due to the diffusion between the core and cladding fluids^12^,^22^. This outcome also reduces the side effects of optical manipulation techniques such as photophoretics-induced biological sample damage^29^. Beyond these intrinsic advantages, the physical and chemical traits of the resulting working fluid after the complete mixing between the core and cladding streams including pH, *n*, density, and viscosity, can be dynamically adjusted by simply controlling the ratio of the flow rates of the core and cladding fluids.

**Figure 5:**
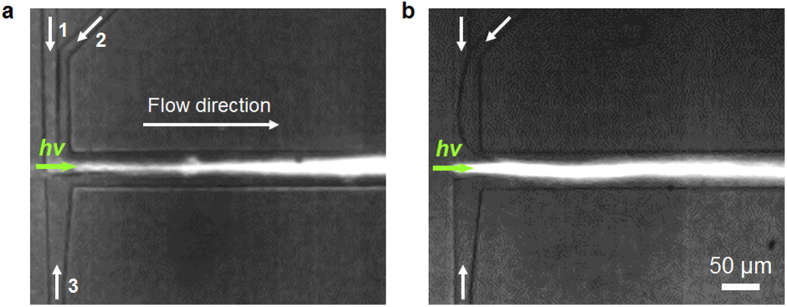
Characteristics of flow and the guided laser beam in the L^2^ waveguide when (**a**) miscible fluids (core/cladding = DMSO/ethanol) and (**b**) immiscible fluids (core/cladding = DMSO/DI water) are used to form the waveguides. Number 1 denotes the core fluid, and 2 and 3 are the cladding fluids.

In a previous study, we numerically evaluated the L^2^ system characteristics based on the balance between the optical force and the viscous drag force^44^. In the actual system, there are two more parameters governing the physics: inertial migration and the interfacial tension force between the core and cladding liquids. The length required for particles to be focused at their equilibrium positions by inertial migration is expressed by^35^


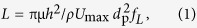


where μ is the fluid dynamic viscosity, h is the channel height, ρ is the fluid density, *U*_max_ is the maximum flow velocity (~1.5 *U*, where *U* is the mean flow velocity), *d*_p_ is the particle diameter, and *f*_L_ is the dimensionless lift coefficient (0.02–0.05 for channel aspect ratios between 0.5 and 2). Inertial migration may be neglected because it plays a nonnegligible role only for channels longer than few centimeters and for particle velocities on the order of a few m/s^35^. However, the interfacial tension force is not negligible. From our calculations, it is of the order of a few nN, which is more than three orders of magnitude higher than the optical force and the viscous drag force (~pN). In general, the interfacial tension force is greater than the surface tension force between the liquid and air. Thus, attempts to reduce the interfacial tension force by changing the combination of the core-cladding liquids are ineffective. We observed that low *n* particles accumulate at the side of the core fluid due to the negative optical radial force when the L^2^ system contains immiscible liquids. However, they do not cross the interface between the core and cladding liquids due to the large interfacial tension force (images are not presented in this paper). Another reason for adopting miscible fluids is that we observed a jiggling shape like lightning arise along the flow direction due to the fluidic instability when the flow velocity was not high enough to reach the Rayleigh stability condition. As shown in Fig. 5, a stable fluidic interface establishes a fairly smooth L^2^ waveguide when miscible fluids (DMSO/ethanol) are used. In contrast, the immiscible fluids DMSO and DI water were found to form an unstable fluidic interface, which usually results in the break-up phenomenon. From the optical perspective, either a graded index (GRIN) waveguide or a step index waveguide can be constructed. In a GRIN waveguide, the width of the guided laser beam expands along the flow direction due to diffusion. In our previous study, the grade profile parameter reflecting the lateral intensity profile was 2 in the GRIN L^2^ waveguide, which means that all modes travel at approximately the same velocity. Accordingly, the modal dispersion phenomenon is prohibited^41^. In contrast, unstable light guiding features hinder the use of step L^2^ waveguides even though they can sustain the optical force influence region longer than the GRIN waveguides.

**Figure 6:**
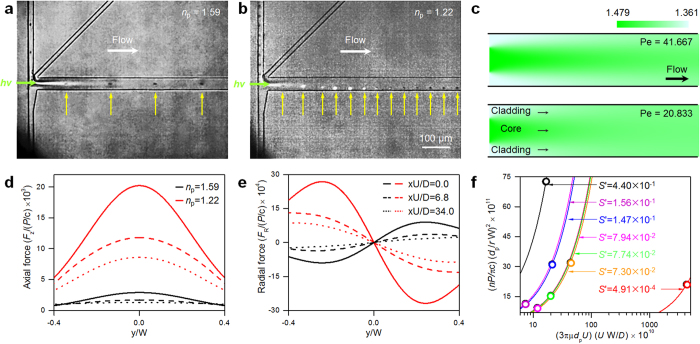
Results for the L^2^ waveguide: trajectories of (**a**) high (d_*p*_ = 5 μm) and (**b**) low (d_*p*_ ~ 4.6 μm) *n* particles, (**c**) *n* profiles through the longitudinal direction for two different Pe values. Numerical calculations of (**d**) optical axial and (**e**) radial forces on high and low *n* particles. The labels of (**d**,**e**) are mutual. (**f**) System performance: experimental results (circles) and numerical predictions (solid lines).

Figure 6 shows some results of our calculations and experiments with the L^2^ waveguide. In general, the L^2^ system is established using a typical hydrodynamic flow focusing geometry^12^,^22^. We observed that in this geometry the laser light leaks at the junction of the core and sheath flows (see the supplementary material). To resolve this problem, the device was modified so that the laser light is coupled simultaneously with the core stream that is sandwiched by the two sheath flows. As for the LS waveguide system, the high *n* particles travel along the core stream while the low *n* particles escape from the core to the cladding liquid region due to the negative optical radial force. As mentioned above, the waveguide fades along the flow direction due to diffusion. Once the particles show the opposite behaviors according to their *n* values, they do not interact with the laser light any further. In this study, we achieved the manipulation of a few micron-sized particles with speeds of up to ~1.64 mm/s at a laser power of 0.415 W, which is quite rapid and efficient for an optical manipulation scheme because of the synchronization of the flow and laser propagation directions. In contrast to systems utilizing an illumination beam which travels and focuses on the focal position and diverges shortly afterward, an elongated optical radial force influence region is obtained by virtue of the waveguide. Results of our simulations of the fluidic conditions are shown in Fig. 6(a,b), for which the convection-diffusion equation was solved in 2D and the *n* profiles were evaluated from the concentration profiles. In Fig. 6(c), Pe = 41.667 and Pe = 20.833 reflect the conditions for Fig. 6(a,b) respectively. By adjusting the flow rates of the core and cladding liquids, the *n* profile along the flow direction can be controlled, which means that the optical force influence region is adjustable. The details of the numerical procedure are described in the Methods section. During the experiments, it was found that the low *n* particles always travel along the lower side wall via negative optical radial force and that they are not pushed into the upper cladding fluid region. These effects arise because the particles co-injected with the core fluid from the upper side slightly overshoot the center of the core due to their inertia, and, as a result, are subject to a negative optical radial force toward the lower side wall. For the calculations of the optical axial and radial forces shown in Fig. 6(d,e) respectively, the longitudinal position, the mean flow velocity, and the diffusion coefficient were included because the optical axial and radial forces are strongly affected by these parameters. Both the axial and radial forces decrease drastically along the longitudinal direction because of the binary diffusion between the core and cladding liquids, which implies that the particles are liberated from the optical force influence region after the optical manipulation at the entrance region. Furthermore, the optical force influence region can be elongated by simply increasing the flow velocity, which results in improved throughput. In order to evaluate the system performance, we adopted the *S* number, which accounts for the ratio of the optical and viscous drag forces^45^. Considering the diffusion effect, the Peclet number (the active advective transport compared to the diffusive transport) was included in the *S*-number, i.e., the *S*′ number (see the Methods section). An identical *S*′ number can be interpreted as the identical optical manipulation characteristics. Since the high *n* particles always pass through the center of the channel due to the positive optical radial force without observable interplay with the optical force and the key mechanism of this system relies on the motion of the low *n* particles, the motions of low *n* particles at different laser powers (0.415–0.426 W), flow velocities (0.708–1.64 mm/s), and particle sizes (4.54–12.5 μm) were analyzed, as shown in Fig. 6(f). The circles indicate the experimental results and the solid lines represent the numerical predictions. A higher *S*′ number was found to ensure more solid separation characteristics, so the experiments were performed mostly at *S*′ numbers between 7.30 × 10^−2^ and 4.40 × 10^−1^. In this study, we achieved low *n* particle manipulation at *S*′ numbers as small as 4.91 × 10^−4^.

## Discussion

We have demonstrated rapid and dynamic particle manipulation with transformative optofluidic waveguides. Integrated liquid-core/solid-cladding (LS) and liquid-core/liquid-cladding (L^2^) system configurations were adopted. The systems have a simple monolithic structure and were found to successfully manipulate particles according to their refractive indices (*n*), regardless of their sizes. The high *n* particles are pulled into the center of the channel by the positive optical radial force whereas the negative optical radial force expels the low *n* particles into the flank region. The present design is working as an on-off system: if the system operating condition is set to be appropriate for the minimum-sized low *n* particle in the sample, the system will let the other larger-sized low *n* particles be removed from the core to the cladding stream because the optical radial force is proportional to the particle size. Previously, it was shown that the particle displacement distance in the lateral direction increases with the particle size even though the viscous drag force increases linearly with the particle size according to the Stokes drag equation^44^. The threshold between the high and low *n* particles can be dynamically adjusted by simply changing the working fluid, which ensures system flexibility and versatility. The LS waveguide was found to have an inherent limitation that influences particle manipulation efficiency. The L^2^ waveguide was found to overcome the issues arising in the LS waveguide. The L^2^ waveguide has additional advantages including the controllability of the optical force influence region and the physical/chemical properties of the resulting working fluid. Numerical calculations were performed that satisfactorily described the physical phenomena. The system performance was evaluated in terms of the *S*′ number and it was found that low *n* particles could be manipulated at *S*′ numbers as small as 4.91 × 10^−4^. In the both LS and L^2^ systems, the pinched region beyond the optical force influence region can be adopted to improve the lateral distance between the high and low *n* particles. Our demonstration of *n*-driven particle manipulation in single-layered optofluidic waveguides provides a new avenue for optical manipulations in the life sciences including bubble removal and malaria diagnostics based on the refractive index^46^.

## Methods

### Microfluidic channel

The PDMS microfluidic channels were fabricated with the conventional soft-lithography process. The widths and heights of the channels were 20 μm and 26 μm respectively for the liquid-core/solid-cladding (LS) system and 50 μm and 20 μm respectively for the liquid-core/liquid-cladding (L^2^) system. A facet-smoothing method was employed to prevent undesirable laser scattering at the interface between the air and the PDMS channel^47^. For the dimensions of the LS waveguide, the light intensity distributions in the cross section were computed using the commercial software, COMSOL Multiphysics. Further details of the numerical analysis and the experimental set-up are provided in the supplementary material.

### Working fluids and test particles

The refractive index (*n*), PDMS compatibility, and light transmittance of several candidate liquids were evaluated with 532 nm laser illumination (see the supplementary material) for the selection of the working fluids. Liquids were transferred to a cuvette with a 1 cm optical path and the light transmittance of each liquid was measured and normalized by the transmittance of water. Every test liquid was found to exhibit moderate PDMS compatibility except chloroform. Dimethyl sulfoxide (DMSO; *n* = 1.48) and ethanol (*n* = 1.36) were used as the working fluids in this study. Mean flow velocities of approximately 550 μm/s and 708–1640 μm/s were used in the LS and L^2^ waveguides respectively. 5 μm polystyrene latex (PSL) particles (*n* = 1.59; Duke Scientific Corp.) and poly-sized (2–20 μm) hollow glass particles (*n* = 1.22; Polyscience, Inc.) were used as the high and low *n* test particles respectively. Even though the *n* value of glass is 1.52, the hollow glass particles are filled with air, and thus their effective *n* value is approximately 1.22. During our experiments, we observed that hollow glass particles that are smaller than ~4 μm in diameter did not exhibit the characteristics of low *n* particles. This phenomenon stemmed from the hollow glass particle manufacturing process; irrelevant to the particle size, they have comparable shell thickness, leading to the *n* discrepancy in terms of the particle size. Alternatively, ultrasound contrast agent microbubbles composed of SF_6_ wrapped with an albumin layer with a thickness of ~100 nm can be considered^48^.

### Computation of the radiation forces on a microsphere

The photon stream method was used. The optical axial and radial forces can be expressed as^40^


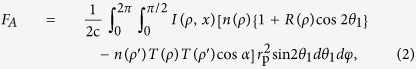



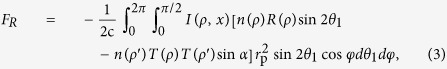


where c denotes the speed of light in free space and *n*(*ρ*) and *n*(*ρ*′) are the *n* values of the surrounding medium at the first (*ρ*) and second (*ρ*′) interfaces respectively. *R* and *T* are the Fresnel reflectance and transmittance respectively. *I*(*ρ*, *x*) is the beam intensity profile given by:


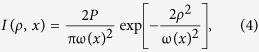


where *P* is the power of the guided laser beam and ω(*x*) is the beam radius along the *x*-direction (i.e., the flow direction).

### Calculation of the refractive index profile in the L^2^ waveguide

The mass conservation equation including both diffusion and convection transport can be formulated as^19^


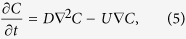


where *C* is the concentration, *t* is time, *D* is the diffusion coefficient (2.00 × 10^−9^ m^2^/s), and *U* is the mean flow velocity. The normalized concentration of either the core or the cladding fluid is expressed as^49^


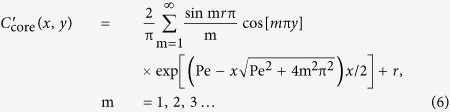



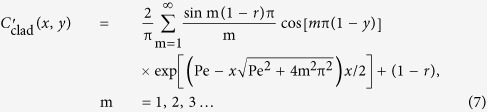


where Pe is the Peclet number defined by *U*W/*D* (W is the width of the channel) and *r* is the position of the interface between the core and cladding fluids *r* = 1/(1 + 2βκ) (β = μ_2_/μ_1_, the dynamic viscosity ratio between the cladding and core flows, and κ = *Q*_2_/*Q*_1_, the ratio of the flow rates of the cladding and core streams). In this study, μ_1_ and μ_2_ are 1.99 mPa · s (DMSO) and 1.08 mPa · s (ethanol) respectively. Equations (6) and (7) are only valid when the flow velocities of all three streams (one core and two sheath flows) are identical. The *n* profile was calculated from the estimated core and the cladding concentrations:


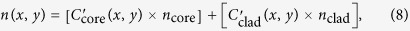


where *n*_core_ and *n*_clad_ are the *n* values of the core and cladding liquids, respectively.

### System performance

The *S* number, the ratio of the optical force and the viscous drag force, is defined by^45^


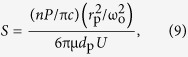


where ω_o_ is the minimum beam waist. In order to account for the diffusion effect in the L^2^ waveguide, the *S*′ number is defined as


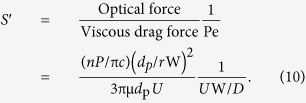


In contrast to the previous study, the minimum beam waist was calculated at the interface between the core and cladding fluids. This definition is plausible here because the higher Peclet number (the active advective transport compared to the diffusive transport) elevates the viscous drag force.

## Additional Information

**How to cite this article**: Lee, K. S. *et al.* Dynamic manipulation of particles via transformative optofluidic waveguides. *Sci. Rep.* **5**, 15170; doi: 10.1038/srep15170 (2015).

## Supplementary Material

Supplementary Information

Supplementary Movie 1

Supplementary Movie 2

Supplementary Movie 3

Supplementary Movie 4

## Figures and Tables

**Figure 1:**
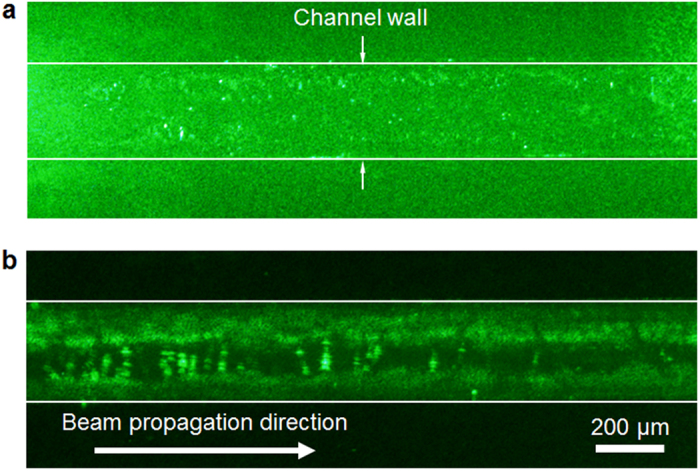
532 nm laser beam propagation through the liquid medium in the PDMS (*n* = 1.41) microchannel: (**a**) No guiding effect is observed when deionized (DI) water (*n* = 1.33) is used as the working fluid. (**b**) The laser beam is guided along the fluidic channel filled with benzyl alcohol (*n* = 1.54) by total internal reflection.
