# Supplementary material for: Dynamic manipulation of particles via transformative optofluidic waveguides
Source: Sci Rep. 2015 Oct 16;5:15170. doi: 10.1038/srep15170 (PMC4607948; doi:10.1038/srep15170)
Supplement: Supplementary Information [file srep15170-s1.doc]

**Dynamic manipulation of particles
via transformative optofluidic waveguides**

Kang Soo Lee, Kyung Heon Lee, Sang Bok Kim, Byung Hang Ha, Jin Ho Jung,

Hyung Jin Sung* and Sang Soo Kim*

*Department of Mechanical Engineering, KAIST*

*291 Daehak-ro, Yuseong-gu, Daejeon 34141, Korea*

**Supplementary Methods**

**Experimental Setup.** Figure S1 shows a schematic diagram of the experimental setup. A continuous wave neodymium-doped yttrium aluminum garnet (Nd:YAG) laser (wavelength, λ = 532 nm; Laser Quantum, UK) was irradiated into the 3× beam expander (Thorlabs Inc., USA) and subsequently focused by 10× objective lens to reduce the laser beam spot size at the focal plane. In order to visualize the experimental process, images were obtained using a high-speed complementary metal-oxide-semiconductor (CMOS) camera (pco. 1200 hs, PCO, USA). A white light emitting diode (LED) source (cool white 5500 K; Mightex, USA) was placed as a light source, and a long-pass filter (cutoff wavelength = 575 nm) was used to block the damage on the CMOS camera due to the residual laser scattering. Two different channels were designed for each experiment: devices with either single inlet or three inlets for liquid-core/solid-cladding (LS) or liquid-core/liquid-cladding (L^2^) systems, respectively. LS was constructed by a single fluid and PDMS polymer as a core and cladding, and widely-used flow focusing geometry was adopted for the L^2^ system configuration. A facet smoothing technique was used to prevent the laser scattering at the interface between the air and PDMS due to the PDMS debris or seamy sidewall during the razor blade cutting. Briefly, a hexahedron box with a top opened was fabricated using slide glasses and epoxy bond. Pre-made PDMS microfluidic channel was attached to a flat PDMS substrate by O­_2_ plasma treatment and placed near the one side wall in the box. Finally, PDMS was poured into the box to fill up the vacancy and baked for the solidification. More details are described in our previous research.^1^ Several candidate liquids were evaluated in terms of their refractive index (*n*), PDMS compatibility, and light transmittance at 532-nm laser illumination for the selection of working fluids (see Fig. S2).

**Formation of Liquid-Core/Liquid-Cladding (L^2^) Waveguide and Its Simple Controllability in Guided Light Mode Manipulation and Optical Switching.** L^2^ waveguide system can be established using a simple hydrodynamic flow focusing geometry. For visualization of the flow and light propagation characteristics, we used green and red dyes, and rhodamine 6G, respectively. As shown in Fig. S3, when the core fluid is sandwiched by the two sheath fluids, light is guided through the core stream via the total internal reflection. By adjusting the flow rate balance between the core and cladding liquids, the cross-sectional area of the core stream is controlled, either single- or multi-mode optical waveguide can be constructed dynamically (see Fig. S4).^2^ Moreover, the lateral position of the core fluid can be controlled when there is a discrepancy between the two sheath flow rates, which has a potential use as an optical switch (See Fig. S5).^2^

**Height of Microfluidic Channel at the Liquid-Core/Solid-Cladding Waveguide.** In the proposed LS system, one of the design considerations is inducing particle motions mainly in the lateral direction, rather than in the vertical direction. It is required to build an optical radial force higher along the horizontal direction. Numerical calculations of the normalized power distribution in a cross section were performed with different channel aspect ratios (H/W): (a) 0.7 (b) 1.0 and (c) 1.3, respectively (see Fig. S6). Because the radial force is proportional to the gradient of the local optical intensity, the aspect ratio larger than unity can build a higher gradient force along the horizontal direction. As a result, Case (c) was chosen as an actual channel design.

**Optical Loss at the Interface between the Core and Cladding Fluids.** Figure S7 shows the light guiding characteristics through the L^2^ waveguide when the optical fiber is assembled to the PDMS microfluidic channel for the light exertion. Some of the light was leaked at the junction of the core and cladding fluids because the converging fluidic shape of the core stream resulted in a locally higher incident angle. Several methods for resolving this issue: reduction of the relaxation region of the core fluid, use of the single mode fiber or focused light. There is a trade-off between the use of optical fiber and focused beam: easy alignment, adaptability, and so forth. In this study, we utilized the focusing lens, however, it can be easily replaced by the optical fiber with simple modifications.

**Supplementary Discussion**

**Enhancement of the Lateral Distance between High and Low *n* Particles Using the Pinched Flow Fractionation (PFF) Geometry.** After the particles are optically manipulated in terms of the *n*, the lateral distance between high and low *n* particles can be enlarged by simply placing the expansion geometry in the downstream, namely pinched flow fractionation (PFF).^3^ As shown in Fig. S8, the high *n* particle, flowing along the center of the channel after the optical manipulation, sustained its lateral position after the expansion region. On the contrary, the low *n* particle flowing along the flank stream was deviated from the center according to the streamline expansion. Even though the PFF geometry does not improve the resolution, it indeed makes an enhancement of the absolute lateral distance which is beneficial to the system flexibility.^4^

**Legends for Supplementary Movies S1-S4**

**Supplementary Movie 1.** Trajectories of the high and low *n* particles in the LS waveguide system.

**Supplementary Movie 2.** Multimode effect on the motion of the high *n* particle in the LS waveguide system.

**Supplementary Movie 3.** Trajectories of high and low *n* particles in the L^2^ waveguide system.

**Supplementary Movie 4.** Enhancement of the lateral distance between high and low *n* particles by the pinched flow fractionation geometry.

**Supplementary References**

1. Lee, K. H., Kim, S. B., Lee, K. S., Jung, J. H., Chang, C. B. & Sung, H. J. Optofluidic purification of blood platelets. *under review* (2015).

2. Wolfe, D. B., Conroy, R. S., Garstecki, P., Mayers, B. T., Fischbach, M. A., Paul, K. E., Prentiss, M. & Whitesides, G. M. Dynamic control of liquid-core/liquid-cladding optical waveguides. *Proc. Natl. Acad. Sci. U.S.A.* **101**, 12434-12438 (2004).

3. Yamada, M., Nakashima, M. & Seki, M. Pinched flow fractionation: continuous size separation of particles utilizing a laminar flow profile in a pinched microchannel. *Anal. Chem.* **76**, 5465-5471 (2004).

4. Lee, K. H., Kim, S. B., Lee, K. S. & Sung, H. J. Enhancement by optical force of separation in pinched flow fractionation. *Lab Chip* **11**, 354-357 (2011).


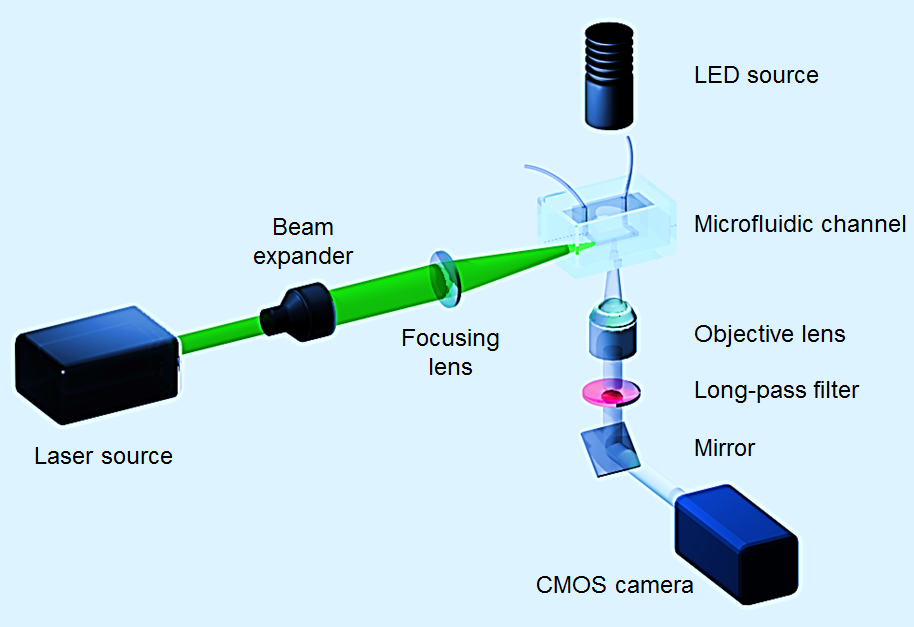


**Supplementary Figure 1.** Schematic diagram of the experimental setup.


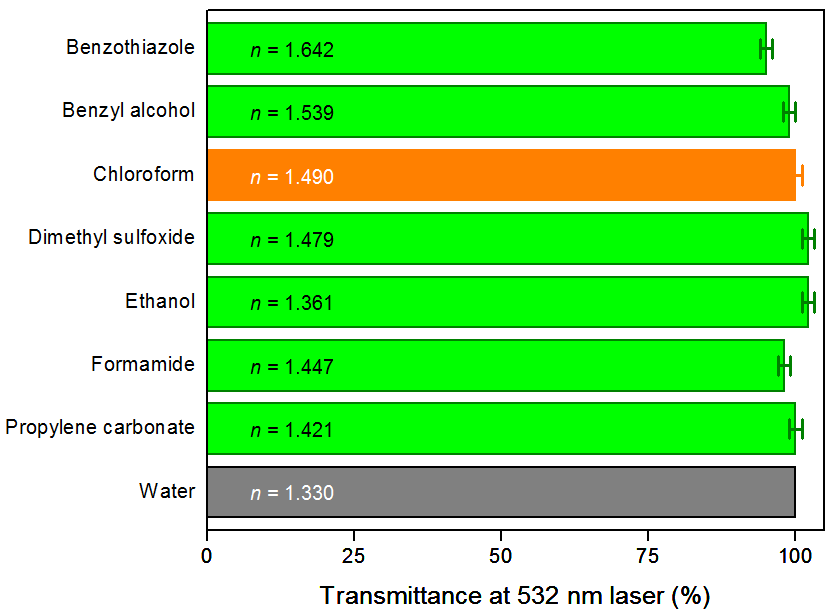


**Supplementary Figure 2.** Properties of several candidate liquids: *n*, PDMS compatibility, and transmittance at 532-nm laser illumination.


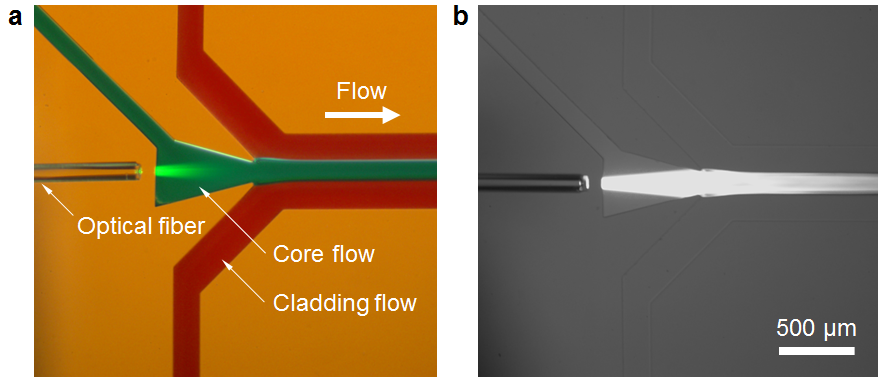


**Supplementary Figure 3.** Construction of the L^2^ waveguide using the hydrodynamic flow focusing geometry: **(a)** flow visualization with green and red dyes and **(b)** guided light along the core stream.


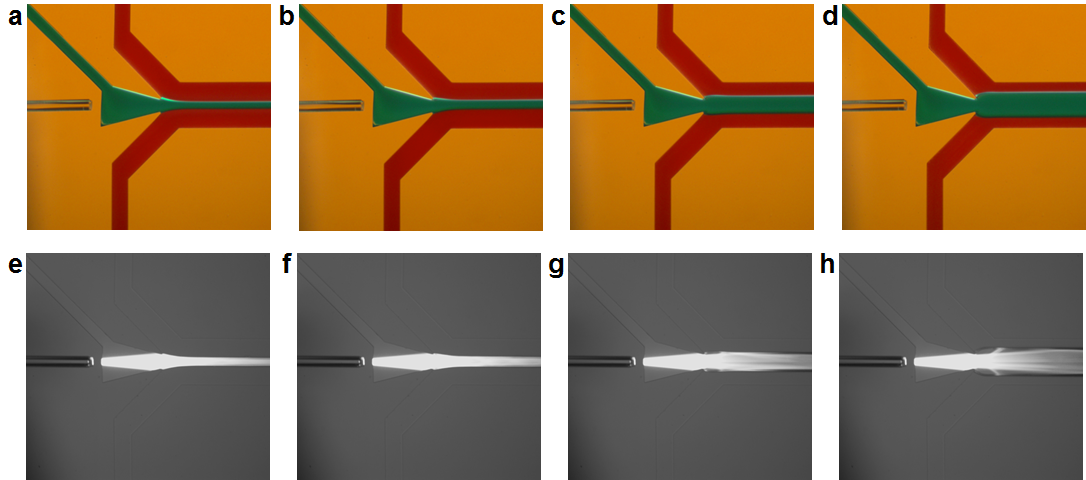


**Supplementary Figure 4.** Control of the number of modes guided along the L^2^ waveguide by adjusting the flow rate ratio between the core and cladding fluids: **(a)**, **(b)**, **(c)**, and **(d)** visualize the fluidic configurations and **(e)**, **(f)**, **(g)**, and **(h)** depict the corresponding guided light shapes. The flow rates are 10/20, 10/15, 10/5, and 10/2, respectively.


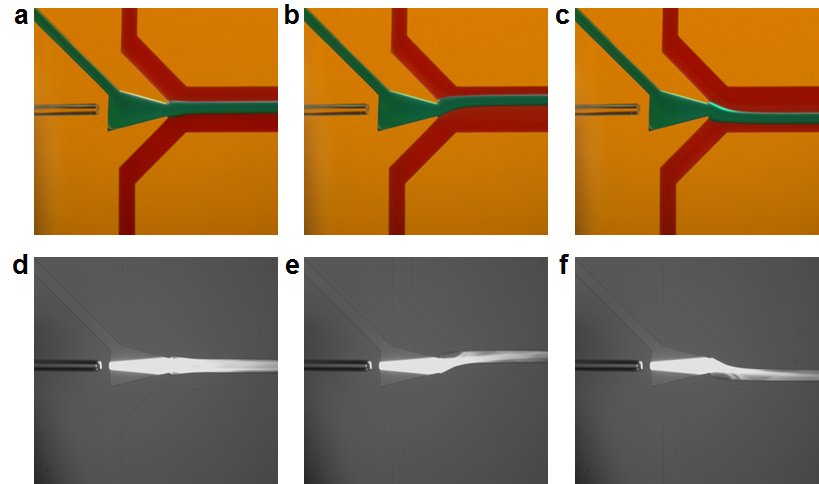


**Supplementary Figure 5.** Lateral position control of the core by adjusting the balance between the two cladding flow rates. **(a)**, **(b)**, and **(c)** visualize the fluidic configurations and **(d)**, **(e)**, and **(f)** depict the corresponding guided light shapes. The flow rates are 10/10/10, 5/10/20, and 20/10/5, respectively.


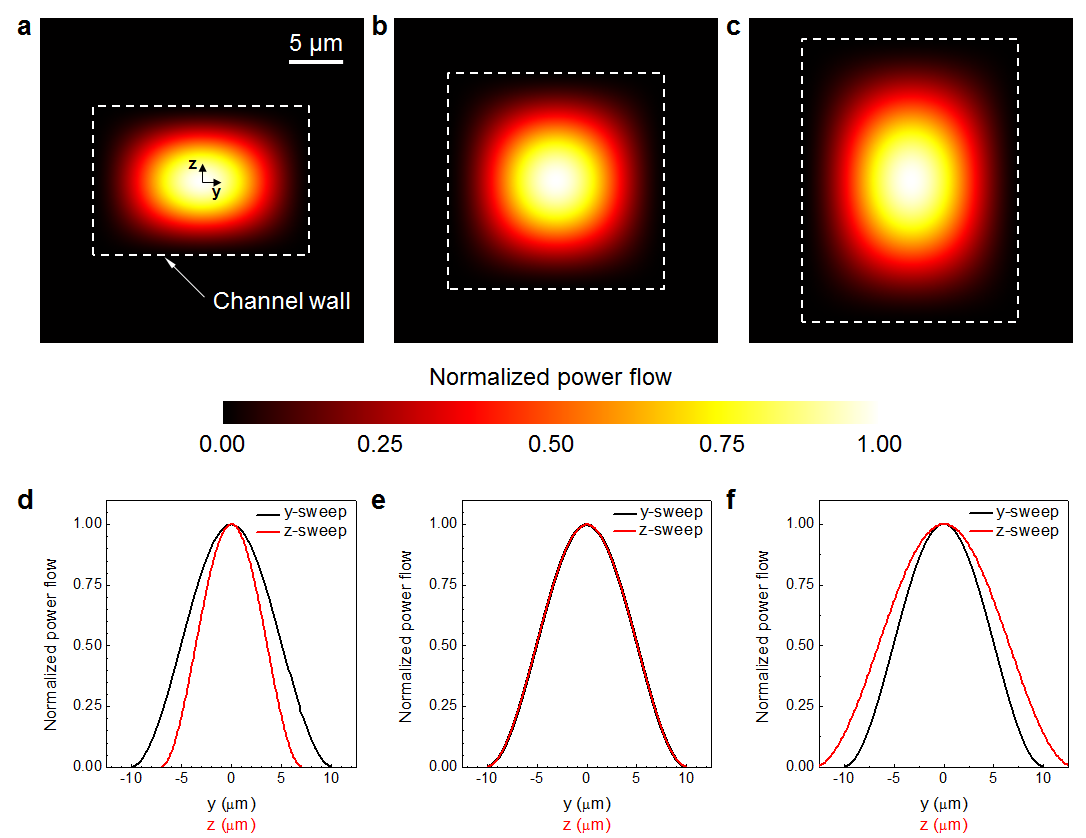


**Supplementary Figure 6.** Numerical calculations in the LS system: normalized power distribution at different channel aspect ratios (H/W) of **(a)** 0.7, **(b)** 1.0, and **(c)** 1.3. **(d)**, **(e)**, and **(f)** describe the corresponding power distributions in the *y*- (lateral) and *z*- (vertical) directions.


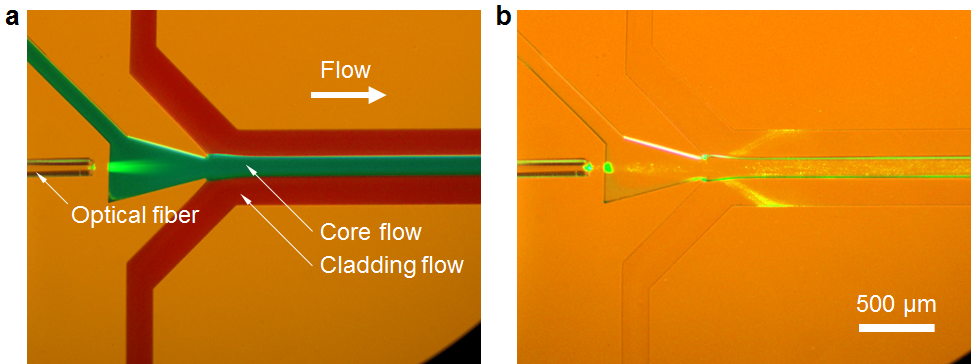


**Supplementary Figure 7.** Light leakage at the junction of the core and cladding liquids.


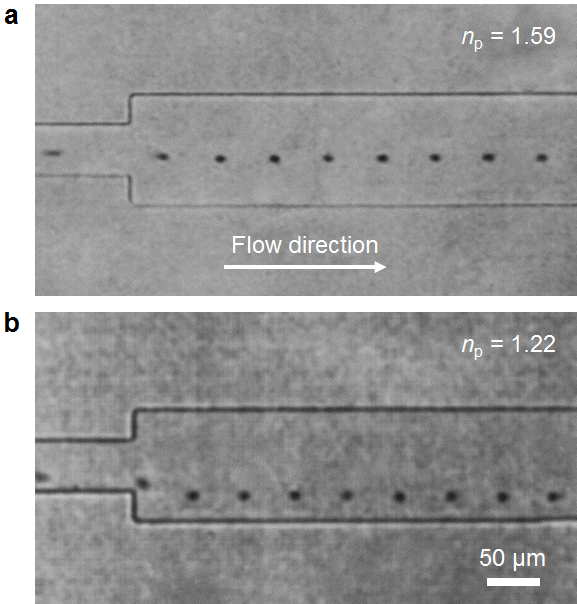


**Supplementary Figure 8.** Particles trajectories at the pinched region: (a) high and (b) low *n* particles.
